# Gadd45b deficiency promotes premature senescence and skin aging

**DOI:** 10.18632/oncotarget.8854

**Published:** 2016-04-20

**Authors:** Andrew Magimaidas, Priyanka Madireddi, Silvia Maifrede, Kaushiki Mukherjee, Barbara Hoffman, Dan A. Liebermann

**Affiliations:** ^1^ Fels Institute for Cancer Research and Molecular Biology, Temple University School of Medicine, Philadelphia, PA, USA; ^2^ Department of Medical Genetics and Molecular Biochemistry, Temple University School of Medicine, Philadelphia, PA, USA

**Keywords:** Gadd45b, senescence, oxidative stress, DNA damage, cell cycle arrest, Gerotarget

## Abstract

The GADD45 family of proteins functions as stress sensors in response to various physiological and environmental stressors. Here we show that primary mouse embryo fibroblasts (MEFs) from Gadd45b null mice proliferate slowly, accumulate increased levels of DNA damage, and senesce prematurely. The impaired proliferation and increased senescence in Gadd45b null MEFs is partially reversed by culturing at physiological oxygen levels, indicating that Gadd45b deficiency leads to decreased ability to cope with oxidative stress. Interestingly, Gadd45b null MEFs arrest at the G2/M phase of cell cycle, in contrast to other senescent MEFs, which arrest at G1. FACS analysis of phospho-histone H3 staining showed that Gadd45b null MEFs are arrested in G2 phase rather than M phase. H2O2 and UV irradiation, known to increase oxidative stress, also triggered increased senescence in Gadd45b null MEFs compared to wild type MEFs. *In vivo* evidence for increased senescence in Gadd45b null mice includes the observation that embryos from Gadd45b null mice exhibit increased senescence staining compared to wild type embryos. Furthermore, it is shown that Gadd45b deficiency promotes senescence and aging phenotypes in mouse skin. Together, these results highlight a novel role for Gadd45b in stress-induced senescence and in tissue aging.

## INTRODUCTION

Gadd45a, Gadd45b and Gadd45g constitute a family of genes, which encode for small (18 kDa) evolutionarily conserved proteins that are highly homologous to each other. Despite marked similarities, these genes are regulated in a differential manner and exhibit functional diversity. They play a pivotal role in regulating diverse cellular functions such as cell cycle control, survival, and apoptosis and are regulated by the nature of the stress stimulus encountered, its magnitude, and the cell type. *Gadd45a, Gadd45b* and *Gadd45g* have been implicated in cell cycle arrest [1–4], DNA repair [5], apoptosis [6], innate immunity [7] and genomic stability [8]. GADD45 proteins have been shown to stimulate the p38-c-Jun N-terminal kinase (JNK) mitogen-activated protein (MAP) kinase pathways in response to stress and thereby sensitize cells to apoptosis, survival or growth arrest [9]. GADD45 proteins were also shown to regulate cell cycle checkpoints in response to genotoxic stress, notably the G2/M checkpoint [10–11]. Furthermore, Gadd45b has also been identified as a transcriptional target of NF-κB, encoding a potent and selective inhibitor of the JNK MAPK pathway and, therefore, of apoptosis [12–13].

Cellular senescence, first identified as a process that limits the proliferation or growth of human cells in culture [14] is now recognized as a crucial tumor suppressor mechanism and formidable barrier to malignant progression [15]. It was also shown to be induced by a variety of potentially oncogenic stimuli, such as telomere shortening, DNA damage, oxidative stress, and oncogene expression [16–17]. MEFs are primary cells with limited life-span, that senesce in culture [18]. The senescence observed in primary MEFs is at least in part due to the stress of them being placed in culture, particularly, hyperoxic culture conditions, which results in accumulation of DNA damage [19–21]. Several studies have revealed a role for Gadd45a in senescence. *Gadd45a* null MEFs do not undergo senescence in response to oncogenic H-ras [22]. Interestingly, in a mouse model of mammary tumorigenesis, loss of *Gadd45a* in the presence of Myc was shown to lead to increased senescence whereas loss of *Gadd45a* in the presence of Ras leads to decreased senescence [23, 24]. Although Gadd45a has been shown to play a significant role in regulating cellular senescence in response to stress, the role of Gadd45b has not been studied. Thus, given the similarities and diversity among the Gadd45 family of genes, it was of interest, to investigate the role of Gadd45b in senescence.

In the current study, we show that mouse embryonic fibroblasts (MEFs) lacking *Gadd45b* exhibit impaired proliferation, a G2 cell-cycle arrest and premature senescence. We also show that *Gadd45b* null cells are more sensitive to hyperoxic stress and have higher levels of DNA damage than *Gadd45b^+/+^* cells. Furthermore, *Gadd45b* null MEFs arrest at the G2/M phase of cell cycle, with impaired G2/M cell-cycle progression, in contrast to other senescent MEFs that arrest at G1. Notably, we show that loss of *Gadd45b* promotes senescence and aging phenotypes in the skin providing *in vivo* evidence for increased senescence in *Gadd45b*^−/−^ mice and suggesting a hitherto unidentified role for *Gadd45b* in regulating stress-induced cellular senescence.

## RESULTS

### Decreased proliferation and premature senescence in *Gadd45b*^−/−^ MEFs

**Figure 1:**
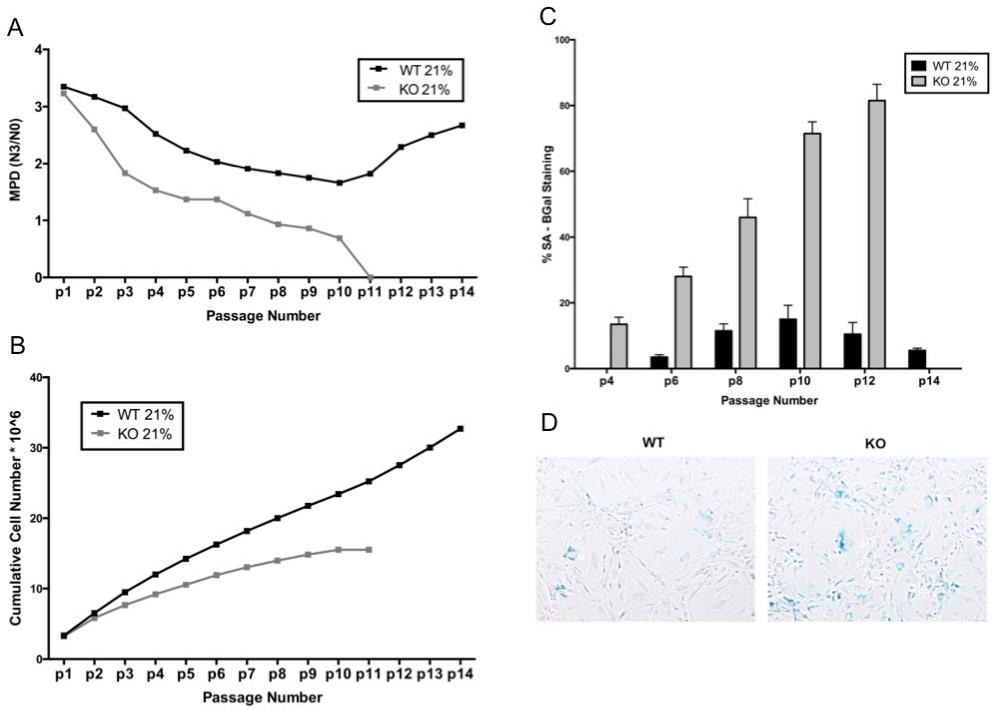
Decreased proliferation and premature senescence of *Gadd45b^−/−^* MEFs **A.** *Gadd45b*^+/+^ (WT - solid black) and *Gadd45b*^−/−^ (KO - solid grey) MEFs were cultured at 21% oxygen continuously for 14 passages. Cells were split every 3 days, and the total numbers of cells were counted and mean population doublings (MPD) were determined. **B.** Cumulative cell number is plotted against passage number. **C.** *Gadd45b*^+/+^ and *Gadd45b*^−/−^ MEFs were stained for SA-β-gal at each passage. SA-β-gal positive cells were counted in at least 10 fields from triplicate plates. A quantification of SA-β-gal positive *Gadd45b*^+/+^ (solid black) and *Gadd45b*^−/−^*(*solid grey) MEFs is shown for different passages. **D.** Representative images of *Gadd45b*^+/+^ and *Gadd45b*^−/−^ MEFs stained for SA-β-gal at passage 9.

*Gadd45b^+/+^* (WT) and *Gadd45b^−/−^* (KO) MEFs were subjected to serial passage using the 3T3 protocol [21, 25, 26] under standard culture conditions, which included atmospheric (20%) oxygen. While the *Gadd45b^+/+^* MEFs exhibited characteristic biphasic growth kinetics seen in mouse embryonic fibroblasts, all *Gadd45b^−/−^* MEFs analyzed showed significantly reduced proliferation (Figure 1A and 1B). This is in striking contrast with *Gadd45a^−/−^* MEFs, which showed increased cell proliferation (Unpublished data). Furthermore, in all MEF cell cultures analyzed, loss of *Gadd45b* was shown to result in premature and increased senescence, as determined by Senescence-associated β-galactosidase (SA-β-gal) staining (Figure 1C and 1D).

**Figure 2:**
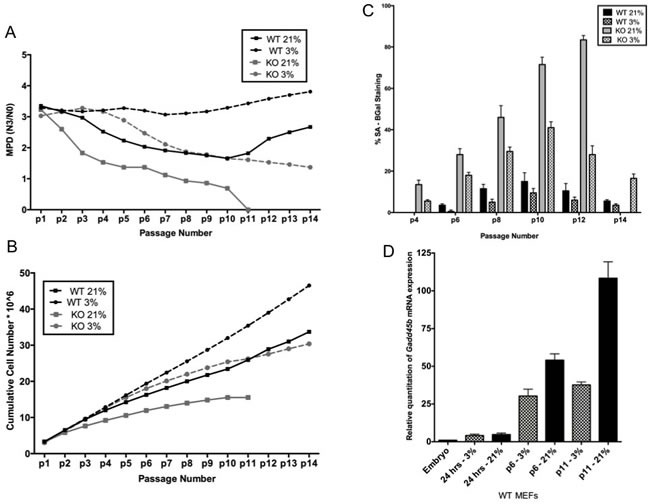
Premature senescence associated with *Gadd45b* deficiency can be rescued, in part, by culture at low oxygen **A.** *Gadd45b*^+/+^ (WT - black), and *Gadd45b*^−/−^ (KO - grey) MEFs were cultured at either 21% oxygen (square) or 3% oxygen (circle) for 14 passages. Cells were split every 3 days, and the total numbers of cells were counted and mean population doublings (MPD) were determined. **B.** Cumulative cell number is plotted against passage number. **C.** *Gadd45b*^+/+^ (black) and *Gadd45b*^−/−^ (grey) MEFs, cultured at either 21% oxygen (solid) or 3% oxygen (dotted) were stained for SA-β-gal at each passage. SA-β-gal positive cells were counted in at least 10 fields from triplicate plates. A quantification of SA-β-gal positive *Gadd45b*^+/+^ and *Gadd45b*^−/−^ MEFs is shown for each passage. **D.** *Gadd45b*^+/+^ MEFs were cultured at either 21% oxygen or 3% oxygen. Total RNA for each passage was analyzed by real time PCR for *Gadd45b* expression using taqman probe as described in Materials and methods. *18S* rRNA probe was used as an internal control.

It is known that primary MEFs are sensitive to oxidative stress in culture [19]. Hence, to test whether exposure to hyperoxia might be a factor in the premature senescence of *Gadd45b^−/−^* MEFs, these MEFs were cultured in the presence of 3% oxygen, which is known to be similar to the physiologic oxygen condition *in vivo*. MEFs were prepared from *Gadd45b^+/+^* and *Gadd45b^−/−^* embryos, and cultured under two different conditions, one at 21% O_2_ and the other under physiologically relevant low oxygen (3% O_2_) conditions. As shown in Figure 2A and 2B, it was observed that culturing cells under physiological oxygen conditions (3% O_2_) resulted in a partial rescue of their ability to proliferate. It should be noted, however, that *Gadd45b^+/+^* MEFs also proliferated faster in 3% O_2_, and the proliferation rate of *Gadd45b^−/−^* MEFs was still much lower than *Gadd45b^+/+^* MEFs. Also, SA-β-gal staining of *Gadd45b^−/−^* MEFs was less at 3% O_2_ compared to staining at 21% O_2_ (Figure 2C) indicating that loss of *Gadd45b* leads to increased sensitivity to oxidative stress and other tissue-culture stressors leading to increased senescence.

It was next asked if oxidative stress modulates *Gadd45b* mRNA expression. Interestingly, we observed that the expression of *Gadd45b* in *Gadd45b^+/+^* MEFs progressively increased with passage numbers (Figure 2D). Also, *Gadd45b* mRNA levels were consistently higher in MEFs cultured at 21% oxygen compared to cells cultured in 3 % oxygen indicating that *Gadd45b* expression is directly correlated to the level of oxidative stress.

### Growth arrest of *Gadd45b*^−/−^ MEFs is associated with defective G2/M cell-cycle progression

**Figure 3:**
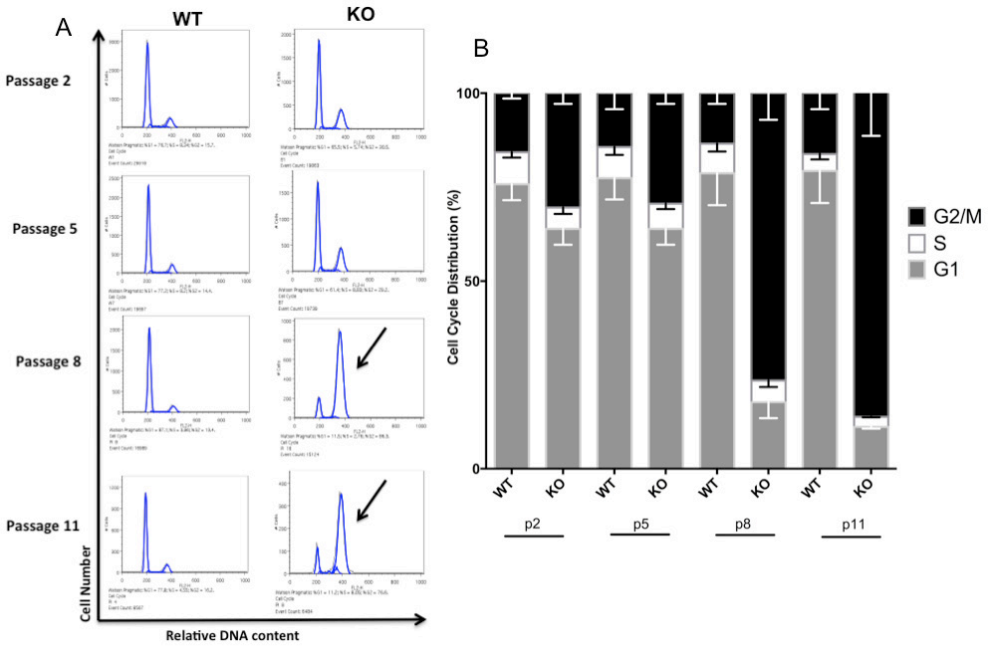
Loss of *Gadd45b* results in an accumulation in G2/M **A.** *Gadd45b*^+/+^ (WT) and *Gadd45b*^−/−^ (KO) MEFs were cultured at 21% oxygen. Subconfluent cultures of cells were harvested at different passages and fixed prior to being stained with propidium iodide. DNA content was analyzed by flow cytometry. Arrow indicates enrichment of G2/M cells in late passage *Gadd45b*^−/−^ cultures. **B.** Quantitation of the fraction of cells in different cell cycle phases was done using FlowJo software. Bar diagram shows the % of cells present in different phases of cell cycle.

**Figure 4:**
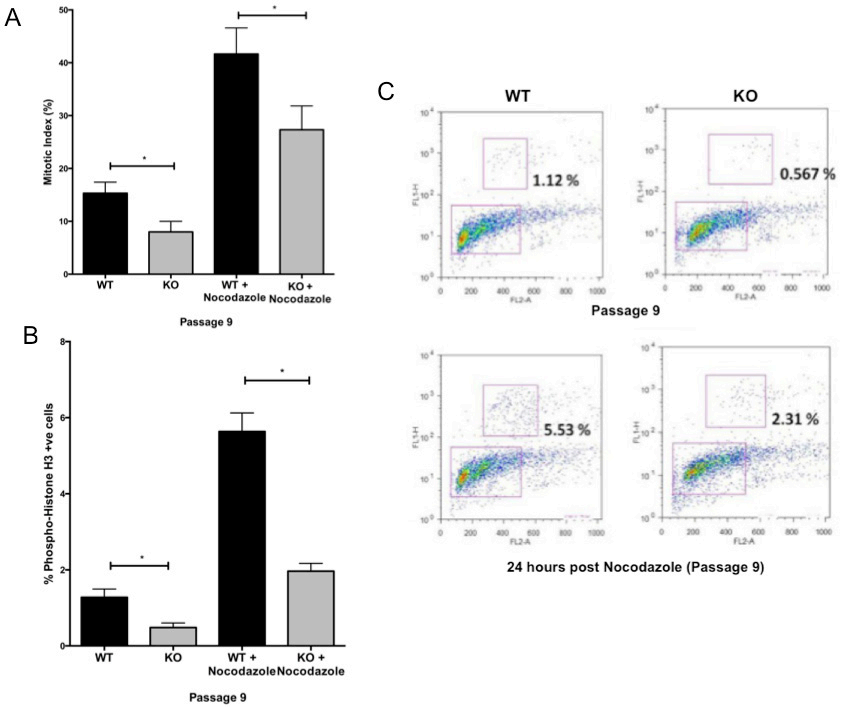
Defective G2/M cell-cycle progression of *Gadd45b^−/−^* MEFs **A.** The mitotic index was determined in *Gadd45b*^+/+^ (WT) and *Gadd45b*^−/−^ (KO) MEFs cultured at 21% oxygen at passage 9. Nocodazol treated cells were used as experimental control. **B.** Number of phosphorylated Histone H3- positive cells in *Gadd45b*^+/+^ and *Gadd45b*^−/−^ MEFs cultured at 21% oxygen (passage 9) using phosphospecific anti-Histone H3 (Ser10) antibodies. Mean values from three independent experiments are shown. **C.** Scatter plots show phospho-histone H3 (y axis) plotted against PI (DNA content, x axis), gated to quantify mitotic (phospho-histone H3 positive) cells (upper box). *P < 0.05.

To determine whether changes in cell cycle control accompany the decline in growth of *Gadd45b^−/−^* MEFs, we compared the cell cycle profiles of *Gadd45b^+/+^* and *Gadd45b^−/−^* MEFs. Surprisingly, *Gadd45b^−/−^* MEFs displayed a gradual increase in the proportion of G2/M cells with increased passage number compared to *Gadd45b^+/+^* MEFs (Figure 3). This finding is in striking contrast to other senescent MEFs, which normally arrest at G1 [27]. To delineate whether *Gadd45b^−/−^* MEFs are arrested in G2 or M phase, we stained *Gadd45b^+/+^* and *Gadd45b^−/−^* MEFs with Hoechst 33342 solution and scored for mitotic stage based on DNA morphology. The mitotic index of *Gadd45b^−/−^* MEFs was lower than that of *Gadd45b^+/+^* MEFs indicating that *Gadd45b^−/−^* MEFs are arrested in G2 phase rather than M phase (Figure 4A). Nocodazole, a microtubule inhibitor used to arrest cells in mitosis, was used as an experimental control.

To further confirm this defect in G2/M cell cycle progression, phospho-Histone H3 staining was performed. Histone H3 is phosphorylated at Ser 10 during M phase, but not at G2 [28]. *Gadd45b^−/−^* MEFs showed less phospho-Histone H3-positive cells than *Gadd45b^+/+^* MEFs (Figure 4B and 4C), indicating that loss of *Gadd45b* in MEFs results in an unexpected defect in G2/M cell-cycle progression.

### Increased p19 Arf -p53-p21 signaling and impaired Cdc2 expression in *Gadd45b*^−/−^ MEFs

**Figure 5:**
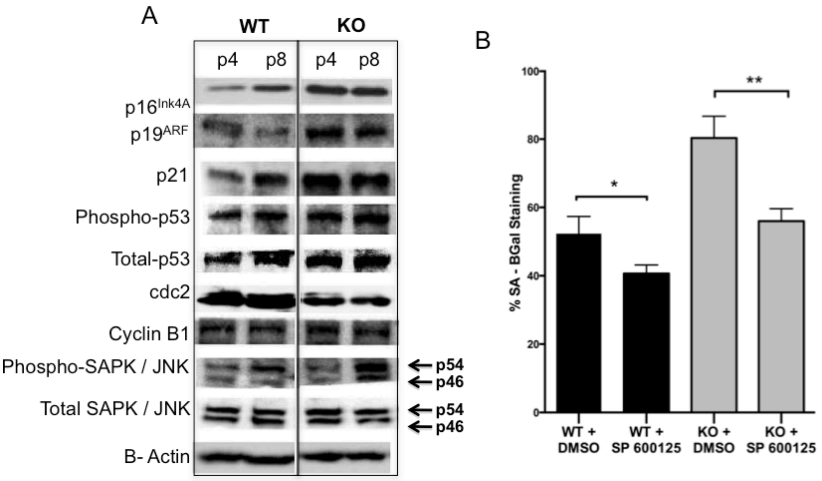
Signaling events involved in *Gadd45b* Senescence **A.** Western blotting analysis of p16^Ink4A^, p19^ARF^, p21, Phospho-p53 (Ser15), Total-p53, cdc2, Cyclin B1, Phospho-SAPK/JNK (Thr183/Tyr185) and Total-SAPK/JNK expression in cell extracts prepared from *Gadd45b*^+/+^ (WT) and *Gadd45b*^−/−^ (KO) MEFs at different passages cultured at 21% oxygen. β-Actin was used as a loading control. **B.** *Gadd45b*^+/+^ (black) and *Gadd45b*^−/−^ (grey) MEFs cultured at 21% oxygen (passage 6) were treated with vehicle (0.1% DMSO) or J*NK inhibitor*, *SP600125* for 24 hours and stained for SA-β-gal 5 days later. SA-β-gal positive cells were counted in at least 10 fields from triplicate plates. A quantification of SA-β-gal positive MEFs is shown. *P < 0.05, **P < 0.01.

Senescence in MEFs was shown to be associated with increased levels of p53 and its downstream target p21 as well as increased levels of p16 and p19^ARF^ [29]. Thus it was of interest to determine the expression levels of p19 Arf -p53-p21 proteins in *Gadd45b^−/−^* MEFs compared to *Gadd45b^+/+^* MEFs. It was found that *Gadd45b^−/−^* MEFs showed increased levels of p16^Ink4A^, p19^ARF^, p21, p53 and phospho-p53 proteins at earlier passage compared to *Gadd45b^+/+^* MEFs, in parallel with reduced growth rate and increased SA-β-gal staining (Figure 5A).

To investigate the mechanism through which Gadd45b regulates proliferation and G2/M cell cycle progression, expression levels of the key G2/M transition complex, Cdc2/cyclin B1 were examined. While the expression of cyclin B1 was comparable between *Gadd45b^+/+^* and *Gadd45b^−/−^* MEFs, intriguingly, a marked reduction in the expression of Cdc2 protein was observed in *Gadd45b^−/−^* MEFs (Figure 5A). Cdc2 has been shown to be essential for G2/M cell-cycle progression in multiple organisms [30]. As shown in Figure 5A, reduced expression of Cdc2 in *Gadd45b^−/−^* MEFs correlated with reduced proliferation indicating that Cdc2 is a potential molecular target for *Gadd45b* regulated stress response. Furthermore, increased phosphorylation of stress-activated protein kinase/Jun-amino-terminal kinase (SAPK/JNK) was also observed in *Gadd45b^−/−^* MEFs compared to *Gadd45b^+/+^* MEFs at late passage (Figure 5A). SAPK/JNK has been shown to regulate p53 dependent senescence [31]. To causally link increased activation of SAPK/JNK in *Gadd45b^−/−^* MEFs to increased tissue culture-induced senescence, *Gadd45b^−/−^* MEFs and *Gadd45b^+/+^* MEFs at passage 6 were treated with JNK specific inhibitor, SP600125 for 48 hours and then stained for senescence-associated β-galactosidase (SA-β-gal) 6 days later. As shown in Figure 5B, inhibition of SAPK/JNK in *Gadd45b^+/+^* and *Gadd45b^−/−^* MEFs led to a decrease in SA-β-gal staining compared to untreated MEFs. Together, these data indicate that *Gadd45b* negatively regulates or limits tissue cultured induced senescence by modulating SAPK/JNK, Cdc2 and senescence signaling.

### Loss of *Gadd45b* results in accumulation of spontaneous DNA damage

**Figure 6:**
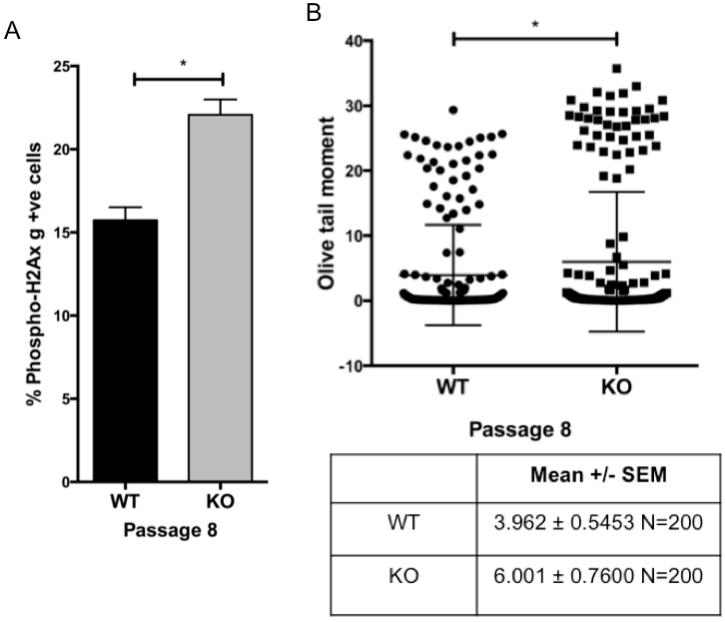
Increased DNA damage in MEFs lacking *Gadd45b* **A.** Numbers of phosphorylated Histone *H2AX* (Ser-139) positive cells in *Gadd45b*^+/+^ (WT) and *Gadd45b*^−/−^ (KO) MEFs cultured at 21% oxygen (passage 8) using phosphor-specific anti-Histone *H2AX* (Ser-139) antibodies. Mean values from three independent experiments are shown. **B.** Dot plot showing DNA comet olive tail moment of 200 *Gadd45b*^+/+^ and *Gadd45b*^−/−^ MEFs cultured at 21% oxygen (passage 8). *P < 0.05. Olive Tail moment is used as a measure of both the smallest detectable size of migrating DNA (reflected in the comet tail length) and the number of relaxed/broken pieces (represented by the intensity of DNA in the tail). All results shown are the mean of three independent experiments ± s.e.m.

Cells with defective mitogen signaling generally accumulate in the G1 phase of cell cycle, whereas arrest in the G2/M phase of cell cycle is frequently indicative of a DNA damage response [32]. The premature senescence induced by hyperoxic stress in *Gadd45b^−/−^* MEFs was associated with accumulation of cells in the G2/M phase of the cell cycle (Figure 3), suggesting that this growth arrest might be associated with higher levels of endogenous DNA damage. To test whether there was increased DNA damage in *Gadd45b^−/−^* MEFs, cells were stained with an antibody that recognizes phosphorylated H2A.X (gH2AX) found specifically at repair foci. As shown in Figure 6A, an increase in phosphorylated gH2A.X positive cells was observed in passage 8 *Gadd45b^−/−^* MEFs compared to *Gadd45b^+/+^* MEFs indicating that loss of *Gadd45b* leads to accumulation of DNA damage. Furthermore, *Gadd45b^−/−^* MEFs showed increased levels of serine 15 phosphorylation of p53 compared to *Gadd45b^+/+^* MEFs (Figure 5A), a modification induced in response to certain forms of DNA damage [33].

To further determine whether the growth arrest of Gadd45b^−/−^ MEFs was associated with accumulation of oxidative DNA damage and directly assess the level of DNA damage on a single-cell basis, we performed comet assay. The assay was done under denaturing conditions to detect both single and double strand breaks and Olive Tail moment was used as a measure of both the smallest detectable size of migrating DNA (reflected in the comet tail length) and the number of relaxed/broken pieces (represented by the intensity of DNA in the tail). As shown in Figure 6B, significantly increased DNA damage, was observed in the Gadd45b^−/−^ MEFs compared to Gadd45b^+/+^ MEFs at passage 8.

Together, these data indicate that loss of Gadd45b leads to an accumulation of DNA damage induced by oxidative stress.

### The increased senescence resulting from *Gadd45b* deficiency is not restricted to tissue culture associated stress

**Figure 7:**
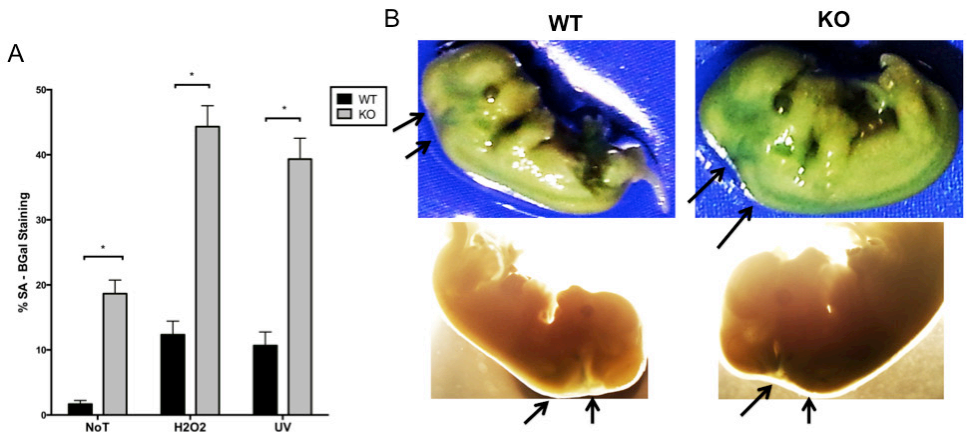
Increased senescence of *Gadd45b^−/−^* mice is not restricted to tissue culture associated stress **A.** *Gadd45b*^+/+^ (WT - black) and *Gadd45b*^−/−^ (KO - grey) MEFs cultured at 21% oxygen (passage 3) were treated with sub-lethal doses of UV-irradiation (30 J/m^2^) or H2O2 (150 uM). Stress-induced senescence (mean numbers of SA-β-gal -positive cells) was determined 7 days after stress. *P < 0.05. **B.** Photograph of SA-β-gal staining of *Gadd45b*^+/+^ and *Gadd45b*^−/−^ E14 embryos. Arrows indicate embryonic regions with strong senescence staining. Strong staining was found in the head and neck regions of all mutant embryos tested (*n* = 6).

Given that environmental stressors such as UV irradiation and oxidative stress have been shown to trigger premature senescence [34], we were interested to determine the effect of these stressors on *Gadd45b^−/−^* MEFs. Early passage *Gadd45b^+/+^* and *Gadd45b^−/−^* MEFs were treated with sub-lethal doses of hydrogen peroxide or UV light. As shown in Figure 7A, an increase in SA-β-gal staining in *Gadd45b^−/−^* MEFs compared to *Gadd45b^+/+^* MEFs was observed for cells treated with either UV or hydrogen peroxide, further confirming that loss of *Gadd45b* leads to an increase in senescence in response to environmental stress. To investigate whether *Gadd45b* deficiency enhanced senescence *in vivo*, we treated E14 embryos from *Gadd45b^+/+^* and *Gadd45b^−/−^* mice with SA-β-gal stain. It is shown that *Gadd45b^−/−^* embryos have increased senescence staining compared to embryos from *Gadd45b^+/+^* mice (Figure 7B), thereby providing *in vivo* evidence that loss of *Gadd45b* leads to increased senescence.

### *Gadd45b* deficiency promotes cellular senescence and accelerated aging in mouse skin

Given the critical role that senescence plays in aging, it was of interest to expand our research on the role of *Gadd45b* in aging using mouse skin as an *in vivo* model system.

**Figure 8:**
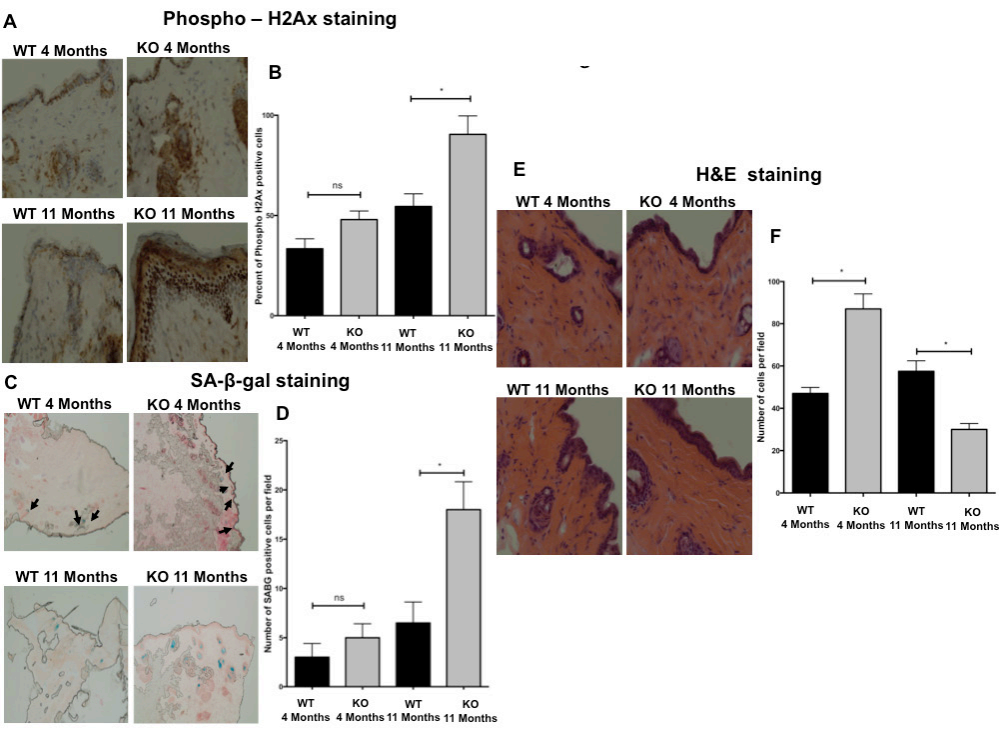
Loss of *Gadd45b* promotes senescence and aging phenotypes in the skin **A.** Representative photomicrographs of immuno-histochemical analysis of phosphorylated histone H2AX (γH2AX) staining of dorsal skin sections from 4 month and 11-month-old *Gadd45b*^+/+^ (WT) and *Gadd45b*^−/−^ (KO) mice. **B.** A quantification of phosphorylated histone H2AX (γH2AX) staining of *Gadd45b*^+/+^ (black) and *Gadd45b*^−/−^ (grey) mice is shown. *P < 0.05. **C.** Representative photomicrographs of SA-β-gal staining analysis of dorsal skin sections from 4 month and 11-month-old *Gadd45b*^+/+^ and *Gadd45b*^−/−^ mice. Arrows indicate skin sections with strong senescence staining. **D.** A quantification of SA-β-gal staining of *Gadd45b*^+/+^ (black) and *Gadd45b*^−/−^ (grey) mice is shown. *P < 0.05. **E.** Representative photomicrographs of histopathological analysis using H&E staining of dorsal skin sections from 4 month and 11-month-old *Gadd45b*^+/+^ and *Gadd45b*^−/−^ mice. **F.** A quantification of purple stained cells in the skin dermis of *Gadd45b*^+/+^ (black) and *Gadd45b*^−/−^ (grey) mice is shown. *P < 0.05.

To investigate the role of *Gadd45b* in DNA damage and aging in skin, dorsal skin sections of 4 month and 11-month-old *Gadd45b^+/+^* and *Gadd45b^−/−^* mice were subjected to immuno-histochemical and histo-pathological analysis. Immuno-histochemical analysis of the well-established marker phosphorylated histone H2AX (γH2AX) [35, 36] was performed to score skin sections for nuclear DNA double-strand breaks. While skin sections from 4 month old *Gadd45b^−/−^* mice showed a slight increase in the number of phospho- histone H2AX (γH2AX) stained cells, skin sections from 11 month old *Gadd45b^−/−^* mice showed a significant increase in the number of phospho- histone H2AX (γH2AX) stained cells compared with their wild type counterparts (Figure 8A and 8B) indicating that *Gadd45b* deficiency leads to increased DNA double-strand breaks in the skin *in vivo*. In order to further characterize this aging phenotype and analyze the role of Gadd45b in modulating senescence in aging skin, senescence associated beta gal staining was carried out. While skin sections from 4 month old *Gadd45b^−/−^* mice showed a slight increase in the number of senescence associated beta gal stained cells, skin sections from 11 month old *Gadd45b^−/−^* mice showed a significant increase in the number of senescence associated beta gal stained cells in the skin dermis (Figure 8C and 8D) compared with their wild type counterparts. Given that DNA damage and senescence play a critical role in the aging of skin, the effect of *Gadd45b* deficiency on skin aging was investigated by histopathological analysis using H&E staining. While skin sections from 4-month-old *Gadd45b^−/−^* mice showed higher number of cells in the dermis, skin sections from 11 month old *Gadd45b^−/−^* mice showed a significant decrease in the number of cells in the dermis (Figure 8E and 8F), compared with their wild type counterparts. Dermal cellularity has been shown to be an essential aging phenotype in skin [37–39]. Thus, taken together, these data indicate that *Gadd45b* deficiency promotes enhanced cellular senescence and DNA damage, as well as a phenotype associated with more advanced aging in skin.

## DISCUSSION

The current study highlights a novel role for *Gadd45b* in the senescence response of mouse fibroblasts to oxidative stress, taking advantage of *Gadd45b^−/−^* mice. Our results show that *Gadd45b* is critical for MEF proliferation and G2/M cell-cycle progression under hyperoxic conditions, since loss of *Gadd45b* leads to premature senescence, defective proliferation and reduced Cdc2 expression. Thus, it seems that under conditions of environmental stress, *Gadd45b* might function to limit the senescence response and maintain the proliferative state of the cells.

As it is known that senescence in MEFs is associated with p19^Arf^ -p53-p21 signaling, we next investigated the expression of these critical cell cycle regulators. Our results show that *Gadd45b^−/−^* MEFs have increased levels of p16, p19^ARF^, p21 and p53 proteins at earlier passage compared to *Gadd45b^+/+^* MEFs. Interestingly, despite the increased expression of critical senescence proteins in *Gadd45b^−/−^* MEFs, these cells do not undergo G1 arrest but rather G2 arrest. It was observed that Gadd45b^−/−^ MEFs accumulated DNA damage, and it is hypothesized that this occurred in the S phase leading to activation of the G2 checkpoint and cells arresting with G2 DNA content. Previous studies have shown that *Mkk7^−/−^* MEFs and *cJun^−/−^* MEFs have impaired proliferation, premature senescence and a G/2M cell-cycle arrest. The G2/M kinase Cdc2 was identified as a molecular target for the MKK7-JNK-cJun-signaling pathway [40, 21]. Given that *Gadd45b* is associated with the MKK7-JNK-cJun pathway, and *Gadd45b^−/−^* MEFs arrest at G2/M, we studied the expression levels of Cdc2. Interestingly, similar to *Mkk7^−/−^* MEFs and *cJun^−/−^* MEFs, *Gadd45b^−/−^* MEFs showed decreased Cdc2 expression indicating that Gadd45b engages the MKK7-JNK-cJun pathway in regulating senescence. Interestingly, blocking JNK activity with the JNK inhibitor SP600125 at passage 6 showed only a modest decrease in SA-β-gal staining. However, it should be pointed out that there is a gradual increase in the senescent cell population with increasing passage number (Figure 1), consistent with JNK inhibition leading to a decrease only in newly senescing cell populations but having no effect on the pre-existing senescent cells.

Similarities between the senescence phenotype of *Gadd45b^−/−^* MEFs and MEFs deficient for genes involved in DNA damage pathways suggested that growth arrest in *Gadd45b^−/−^* MEFs might be attributable to DNA damage accumulation. To test this, we cultured *Gadd45b^−/−^* MEFs under conditions which limit oxidative DNA damage, the major environmental insult cells experience during conventional culture conditions [19]. Our results show that culturing cells under more physiological oxygen conditions results in partial rescue of their ability to proliferate, thereby demonstrating that *Gadd45b^−/−^* MEFs have increased sensitivity to oxidative stress. It should be noted that *Gadd45b^+/+^* MEFs also proliferated better in 3% oxygen; however, there was still a difference between *Gadd45b^+/+^* and *Gadd45b^−/−^* MEFs. Both *Gadd45b^+/+^* and *Gadd45b^−/−^* MEFs had high levels of DNA damage and gH2AX-containing repair foci in their nuclei, but the number of repair foci and the amount of DNA damage were significantly higher in the *Gadd45b^−/−^* MEFs. Previous reports have shown that normal mouse cell senescence occurs as a consequence of the accumulation of DNA damage resulting from hyperoxic culture conditions [20–21]. Our data provide an important extension of this notion, showing for the first time that *Gadd45b* plays a critical role in protecting MEFs from oxidative stress and limiting the DNA damage and senescence response. In addition to oxidative stress, other environmental stresses such as UV irradiation and hydrogen peroxide also trigger premature senescence in *Gadd45b^−/−^* MEFs compared to *Gadd45b^+/+^* MEFs.

Interestingly, increased senescence was observed in E14 embryos from *Gadd45b*^−/−^ mice compared to wild type providing *in vivo* evidence for increased senescence in *Gadd45b*^−/−^ mice. Given that the temporal expression patterns of *Gadd45* genes are dynamic and differential during mouse embryonic development [41] it is hypothesized that *Gadd45b* might function to limit the senescence response to various stimulants involved in embryonic development, a response that is compromised in *Gadd45b*^−/−^ mice leading to increased senescence. Furthermore, transgenic mice for tamoxifen-induced *Dicer* ablation [42] as well as hypomorphic mutation of *Brca1 (Brca1*∆*11/*∆*11)* [43], genes that regulate DNA damage [44], also showed increased senescence staining in mutant embryos compared to wild type. While this work was in progress, two landmark studies found evidence for the presence of senes­cent cells in mouse and human embryos showing that senescence is a normal programmed mechanism that plays instructive roles in tissue remodeling and development [45–46]. They identified non-dividing SA-β-gal-containing cells in different regions of the embryo that also expressed high levels of p21. It will therefore be interesting to further investigate the senescence- limiting effect of *Gadd45b* on normal embryonic development, characterize the senescence distribution among different tissues in the embryos and analyze the senescence pattern during the various stages of embryonic development.

We also show that loss of *Gadd45b* promotes aging phenotypes in mouse skin including increased DNA damage, increased senescence and decreased dermal cellularity. Similar aging phenotypes have been reported previously, including mice with chronic activation of p53 (p53^TSD/−^ mice with two phosphomimetic mutations (T21D and S23D) [47], *Sod2* deficient mice [48] and *Mdm2* deficient mice [49]. It will be of interest to determine whether there is crosstalk between *Gadd45b* regulated molecular pathways that prevent premature aging and molecular pathways regulated by these other proteins.

Finally, our findings raise several interesting questions warranting further investigation: These include: how *Gadd45b* regulates senescence driven by other stressors; how *Gadd45b* interfaces with different signaling pathways in response to distinct stressors; what role *Gadd45b* expression or the lack of it plays in the senescence response of human cells; what is the effect of loss of *Gadd45b* on aging in other organs and the entire mouse life span; and do other Gadd45 genes (i.e., Gadd45a and Gadd45g), either separately or in combination with Gadd45b, regulate stress induced senescence. Several other genes including *Mkk7* [40], *cJun* [21], *Brca1* [43], *Id1* [50], *Hus1* [51], *Jnk* [31], *Ku80* [52], *Polmu* [53], *Vhl* [54], *Dicer* [42] and *TGFβ* [20] have been observed to limit tissue culture-induced senescence similar to *Gadd45b*, where their loss resulted in premature senescence. Thus, it is of interest to determine whether there is crosstalk between *Gadd45b* regulated molecular pathways that protect cells from undergoing tissue culture-induced senescence and molecular pathways regulated by these other proteins. Notably, the observation that *Gadd45b* loss results in premature senescence is in contrast to *Gadd45a* KO MEFs that were observed to escape senescence (Unpublished data). Thus, it will also be of interest to compare and contrast *Gadd45b* signaling to *Gadd45a* signaling in MEFs, where loss of *Gadd45a* results in escape from tissue cultured senescence. Current research is targeted at addressing these interesting issues.

In conclusion, the results obtained indicate that GADD45 proteins differentially modulate stress- and tissue culture-induced cellular senescence, providing the impetus to further investigate the role of GADD45 proteins in senescence under normal physiological conditions as well as various pathological conditions.

## MATERIALS AND METHODS

### Animals

Mice were maintained in a temperature and humidity-controlled environment at Temple University's Health Science campus animal facilities following the guidelines of Institutional Animal Care and Use Committee (IACUC) of Temple University. Mice genotype was confirmed by PCR using specific primer sets for *Gadd45b* and the neomycin phosphotransferase reporter gene each time the litters were produced. MEFs were obtained from 13.5-day WT or *Gadd45b^−/−^* sibling embryos.

### Cell culture

MEFs were grown in Dulbecco's modified Eagle's medium (DMEM) supplemented with 10% fetal bovine serum in 3% O_2_/5% CO_2_ for 2 days, then harvested, viably frozen, and labeled as passage 0. For 3T3 assays, MEFs were seeded at 3×10^5^ cells per 10 cm plate, trypsinized after three days, counted and re-seeded at the same density. For growth at low oxygen levels, MEFs were grown in a humidified hypoxia chamber that was flooded with a gas mixture of 92% Nitrogen, 3% Oxygen, 5% Carbon Dioxide. Cells were counted in triplicate. The number of cells obtained on day3 (N3) was divided by the initial cell number (N0 = 3X10^5^) and plotted as growth rate (N3/N0). The increase in the population doubling level (ΔPDL) was calculated according to the following formula: ΔPDL = log (Nf/N0)/log 2, where N0 is the initial number of cells (3X10^5^) and Nf is the final number of cells.

### UV irradiation and hydrogen peroxide treatment

UV irradiation (50 J/m2) of cells was carried out with a Stratalinker (Fisher Scientific) adjusted to UV-C irradiation. The cells were washed with phosphate-buffered saline before irradiation in the absence of any medium. Following irradiation, cells were supplemented with culture medium for 7 days and analyzed for SA-β-gal activity. Oxidative stress was induced by sub-cytotoxic levels of hydrogen peroxide (150 μM for 4 hours) and then cells were recovered in normal medium for 7 days and analyzed for SA-β-gal activity.

### Senescence-associated β-galactosidase (SA-β-gal) assay

Senescent cells were detected by staining for beta-gal using X-Gal (Cell Signaling, Danvers, MA). Stained cells were visualized using an Olympus inverted microscope with digital imaging (Leica MZ16 stereomicroscope) and images were captured with a QImaging 5.0 RTV digital camera. A total of 300-400 cells were evaluated to assess the percentage of SA- β Gal positive cells. Similarly, whole-mount embryo SA- β Gal was also detected following overnight fixation and incubation with X-gal for 4-6 h.

### Western blotting

Cells were incubated in cell lysis buffer (no. 9803; Cell Signaling Technology, Danvers, MA) followed by centrifugation at 13,000 rpm for 10 minutes. Supernatants were collected, and protein concentrations were determined using the Bradford Assay (Bio-Rad, Hercules, CA). Protein lysates were electrophoresed on Tris-glycine SDS polyacrylamide gels and transferred onto Immobilon-P membrane. Incubation with antibodies was performed according to Cell Signaling Technology-recommended procedures and proteins were visualized using ECL (Pierce). p16^Ink4A^, p19^ARF^, p21, Total-p53, cdc2 were from Abcam. Phospho-p53(Ser15), Cyclin B1, Phospho-SAPK/JNK(Thr183/Tyr185), Total-SAPK/JNK and β-Actin were from Cell Signaling Technology.

### RNA extraction and quantitative polymerase chain reaction

RNA was extracted from samples using RNeasy kit (Qiagen) according to manufacturer's protocol. Reverse transcriptase polymerase chain reaction (RT-PCR) was performed to convert RNA to c-DNA using TaqMan Reverse Transcription reagents (Applied Bio systems) according to manufacturer's protocol. c-DNA was then used to run the real time polymerase chain reaction analysis (qRT-PCR) in a StepOne Real Time PCR machine (Applied Bio systems). Taqman probes used for this study, purchased from Life Technologies, are the following: - Mm00435123_m1 (mouse *Gadd45b*), and Mm04277571_S1, for 18s, used as an endogenous control.

### Flow cytometric cell cycle analysis

Cells were collected at the indicated time points and fixed in methanol. Prior to analysis, cells were treated with a solution containing 10% propidium iodide (500 μg/ml), 5 mg/ml 10% RNase A and 80% 1× phosphate-buffered saline (PBS) plus 1% FBS, and incubated at 37°C for 30 min. The cells were analyzed with a FACSCalibur (BD) flow cytometer, and the data were analyzed using FlowJo analysis software (Tree Star). To determine cell cycle distributions in the G2 and M phases, cells were harvested with trypsin, stained using anti phospho-histone H3 - AlexaFluor^®^ 488 antibody on ice for one hour in the dark, followed by staining using PI/RNase solution for 30 minutes at room temperature in the dark per the manufacturer's instructions (#FCCH025103, EMD Millipore Corporation, Ballerica, MA). The samples were analyzed by a FACScan flow cytometer (Becton-Dickinson), with FlowJo analysis software (Tree Star).

### Immunofluorescence and mitotic index analysis

Hoechst 33342 stock solution (Molecular Probes) was diluted 1:100 in H_2_O. The medium was aspirated from cells grown on cover slips and the cells were rinsed three times with PBS. The cells were incubated in the diluted Hoechst solution for 15 min at room temperature. After labeling, cells were washed with PBS and imaged under an EVOS™ Digital Inverted Fluorescence Microscope. The mitotic index, i.e., the percentage of cells in mitosis was determined by counting at least 300 cells in three independent experiments. The mitotic index was determined by fluorescence microscopic analysis of Hoechst 33342 stained nuclear morphology.

### Alkaline comet assays

Comet assays were performed using the Trevigen Comet Assay Kit (4250-050-K) as per the manufacturer's instructions. Cells (1000) were mixed with low melt agarose, spotted onto slides, lysed, and electrophoresed under denaturing conditions at 4 degree C. DNA was stained with SYBR green and images were captured at 20x magnification using an EVOS™ Digital Inverted Fluorescence Microscope. Images were saved as bitmap files and olive tail moments were calculated using TriTek CometScoreTM Freeware v1.5.

### Histo-pathological and immuno-histochemical evaluation of skin samples

Dorsal skin samples from euthanized mice were fixed in 10% buffered formalin for 24 h, transferred to 70% ethanol, embedded in paraffin, and sliced. The sections were stained with hematoxylin and eosin (H&E) for histological assessment.

Optimal Cutting Temperature compound (OCT)-embedded samples were cut into 10 μm sections. Sections were fixed in 10% buffered formalin, permeabilized with 0.5% triton-X, blocked with 4% donkey serum/1% BSA in PBS solution and incubated with anti-γH2AX (NB100-79967, Novus Biologicals, 1:500) overnight at 4°C, followed by incubation with Alexa 555 donkey anti-rabbit (Invitrogen, 1:750) for 1 h at room temperature. Sections were mounted with Prolong Gold with DAPI (Invitrogen).

OCT-embedded skin sections were processed for SA-β-gal staining using Senescence Detection Kit (BioVision, Mountain View, CA, USA). Sections were counterstained with nuclear fast red and visualized by brightfield microscopy.

### Statistical analysis

Data are presented as mean ± s.e.m. Unless indicated all analyses were done by using Student's t test *P < 0.05, **P < 0.01 and ***P < 0.001.
